# Neonatal Abstinence Signs during Treatment: Trajectory, Resurgence and Heterogeneity

**DOI:** 10.3390/children11020203

**Published:** 2024-02-05

**Authors:** Jennifer S. Miller, Henrietta S. Bada, Philip M. Westgate, Thitinart Sithisarn, Markos Leggas

**Affiliations:** 1 College of Nursing, University of Tennessee, Knoxville, TN 37996, USA; wsmith6@utk.edu; 2 College of Medicine, University of Kentucky, Lexington, KY 40536, USA; tsith2@uky.edu; 3 College of Public Health, University of Kentucky, Lexington, KY 40536, USA; philip.westgate@uky.edu; 4 Division of Pharmaceutical Sciences, St. Jude Children’s Research Hospital, Memphis, TN 38105, USA; mark.leggas@stjude.org

**Keywords:** neonatal abstinence syndrome (NAS), neonatal opioid withdrawal syndrome (NOWS), CNS/GI withdrawal signs trajectory, Finnegan neonatal abstinence scoring tool (FNAST), gut–brain axis, length of treatment (LOT), withdrawal sign resurgence

## Abstract

Neonatal abstinence syndrome (NAS) presents with a varying severity of withdrawal signs and length of treatment (LOT). We examined the course and relevance of each of the NAS withdrawal signs during treatment in a sample of 182 infants with any prenatal opioid exposure, gestational age ≥ 35 weeks, without other medical conditions, and meeting the criteria for pharmacological treatment. Infants were monitored using the Finnegan Neonatal Abstinence Scoring Tool. Daily mean Finnegan scores were estimated using linear mixed models with random subject effects to account for repeated withdrawal scores from the same subject. Daily item prevalence was estimated using generalized estimating equations with a within-subject exchangeable correlation structure. The median LOT was 12.86 days. The prevalence of withdrawal signs decreased from day one to day three of treatment. However, certain central nervous system (CNS) and gastrointestinal (GI) signs showed sporadic increases in prevalence notable around two weeks of treatment, accounting for increases in Finnegan scores that guided pharmacotherapy. We question whether the resurgence of signs with a prolonged LOT is mainly a consequence of opioid tolerance or withdrawal. Monitoring CNS and GI signs throughout treatment is crucial. Future studies directed to better understand this clinical phenomenon may lead to the refining of NAS pharmacotherapy and perhaps the discovery of treatment alternatives.

## 1. Introduction

The incidence of infants born experiencing neonatal abstinence syndrome (NAS) has increased with the continued rise in opioid use during pregnancy [1]. Between 2012 and 2016, the incidence of NAS rose from 4.6 to 7.3 per 1000 in in-hospital live births [2,3]. Because most reported NAS cases were associated with prenatal opioid exposure, NAS has been referred to recently as neonatal opioid withdrawal syndrome (NOWS) [4]. However, withdrawal manifestations could not be attributable solely to opioid exposure, since opioids are often used with tobacco, alcohol, and/or other drugs, legal or illicit. Thus, NAS would be a more appropriate term [5].

Infants with NAS often have a prolonged length of stay that is associated with the duration of pharmacological treatment [6,7]. The syndrome occurs in a spectrum of severity with its onset dependent on exposure type [6,8]. In a seminal article by Desmond, the careful clinical monitoring of infants with withdrawal manifestations, primarily due to heroin, methadone, or barbiturates, revealed different phases or variation in the clinical course of NAS [8]. Some infants may have minimal transient signs of withdrawal undistinguishable from typical newborn behavior. Others may have a delayed onset of a few days of overt signs, a step-wise increase in severity, an intermittent appearance of signs, or a bi-phasic course that shows improvement at around two weeks followed by the resurgence of signs that may last for several weeks to months [8]. With the similarity of newborn withdrawal manifestation to those of drug-dependent adults as assessed by a scoring tool [9], Finnegan and colleagues created a 21-item scoring tool to identify withdrawal signs involving the central nervous system (CNS), as well as the metabolic, vasomotor and/or respiratory (MVR), and gastrointestinal (GI) systems [10]. The identification of each observed sign needed specific non-pharmacological interventions [11]. The Finnegan neonatal abstinence scoring tool (FNAST) is now the most widely used tool for NAS assessment, and yet, it is criticized for its length, complexity, and subjectivity [6]. The complexity of the FNAST led to the development of alternatives [12,13,14,15] and a recommendation to abandon the FNAST altogether [16].

The proposed alternatives to the FNAST [17] have included the shortened FNAST [12,13,14,15], Eat, Sleep, Console (ESC) [18], skin conductance [19], infant pupillary diameter [20], and acoustic characteristics of the infant cry [21]. The shortened tools, as summarized by Miller et al. [22], were meant to either “optimize” covariance with the FNAST or predict the need for pharmacological treatment.

Not all infants with NAS require pharmacologic treatment. Roughly 40–80% will require medication, usually with morphine [6,23], with the dosage titrated based on the severity of the clinical signs as guided by the total FNAST score (FS), the sum of items’ scores during each FNAST assessment. The response to therapy or length of treatment (LOT) varies from a few days to several weeks, or even months, but it is unclear whether the treatment response relates to the differences in the patterns or course of NAS as described several decades ago [8], the type of pharmacotherapy notwithstanding.

The importance of individual FNAST items at the point of treatment initiation has been studied [22]. However, the trajectory of each of the infant’s signs as indicated by FS, assessed at 3 to 4 h intervals over the course of treatment, is unknown. We aimed to identify what we may miss if we forgo the FNAST altogether. We therefore determined how relevant each FNAST item is after infants start pharmacological treatment and how this relates to serial FS determinations. Specifically, we looked at mean FS over time and prevalence trajectories to determine which items or signs seem to diminish with treatment, which items do not, and which items tend to still be important weeks after treatment initiation. We explored if the LOT is associated with item prevalence trajectories. Knowledge of the trajectories of an infant’s NAS manifestations during pharmacological treatment could be useful as facilities decide which alternatives to consider by solidifying the understanding of the pertinent manifestations to be included in any alternative assessment [24]. By identifying specific signs that are commonly present in infants requiring long-term treatment for NAS, there is a potential for personalized clinical management focused specifically on those important signs.

## 2. Materials and Methods

### 2.1. Study Methods

This study was approved by the Institutional Review Board at the University of Kentucky. Using electronic medical records, we collected data on infants diagnosed with NAS between 2018 and 2020 using ICD-10 codes P96.1 or P04.9. The infants for this study were a part of a retrospective cohort (*n* = 369) to evaluate the different shortened or simplified FNAST [22]. Only those that met treatment criteria (*n* = 182) were included in this current study in addition to the following: any prenatal opioid exposure, gestational age ≥ 35 weeks, FS assessed during hospital stay, and with no other medical conditions. The criteria for initiating treatment were an FS score of ≥8 over three consecutive time periods or an FS score of ≥12 over two consecutive time periods. Infants had FS assessed every 3 h by trained registered nurses. The standard hospital treatment protocol included the initiation of oral morphine at 0.05 mg/kg per dose given every 3 h. If there was no response after 12 to 24 h, there were subsequent dose escalations by 0.0125 mg/kg/dose until a maximum dose of 0.12 mg/kg/dose was reached. In cases with no improvement, adjunctive treatment with phenobarbital or clonidine was given. The FS guided the dosage titration. All infants received non-pharmacological interventions to mitigate withdrawal signs; rooming-in and breastfeeding were encouraged [11,25,26,27].

### 2.2. Statistical Analyses

Using the dataset collected from a previous study that addressed the potential clinical utility of four shortened scores relative to the FNAST [22], this study calculated days of treatment as the time elapsed since treatment initiation in a more refined approach. All available observations during the first 24 h post-treatment initiation period were considered the first day of treatment. The second day of treatment would be greater than 24 h and up to 48 h post-initiation and so on. The analyses included the daily mean FS scores and item prevalence through the first 36 days of treatment, corresponding approximately to the 95th percentile of our sample’s LOT. For comparisons, we created two groups of infants based on our sample’s median LOT and compared their mean scores and item prevalence. We then asked if the LOT was associated with item prevalence trajectories.

To estimate daily mean scores and to obtain corresponding 95% confidence intervals, linear mixed models with random subject effects were used to account for repeated scores from the same subject [28]. To estimate daily item prevalence and to obtain corresponding 95% confidence intervals, generalized estimating equations with a within-subject exchangeable correlation structure were used [29]. The 95% confidence intervals are presented in the figures for estimates to provide a measure of precision, as the results were meant to be descriptive with respect to the remaining infants on the given day. Confidence intervals were also presented for the plots comparing the two groups of infants by LOT ≤ 13 and >13 days. For convenience, we presented a select few within the manuscript, including two plots of prevalence corresponding to a combination of select CNS and GI signs. Figures were created using base graphics in R 4.2.1 [30,31] and PRISM GraphPad [32].

Since mean FS scores notably decreased from the first to the third day of treatment, an exploratory analysis was done to determine how strongly mean daily FS during the first three days of treatment were associated with the LOT by using correlations and multiple linear regression. Specifically, bivariate associations were assessed using Pearson’s correlations between the daily mean scores and the natural log of the LOT. To determine the most significant of the first three treatment days for predicting the natural log of the LOT and how strongly these days are associated with the LOT, we used multiple linear regression and backward elimination at the 0.05 significance level with two-sided *t*-tests. The natural log of the LOT was used to ensure statistical assumptions were met. Analyses were conducted in SAS version 9.4 [33].

## 3. Results

### 3.1. Sample of Infants Studied

**Figure 1:**
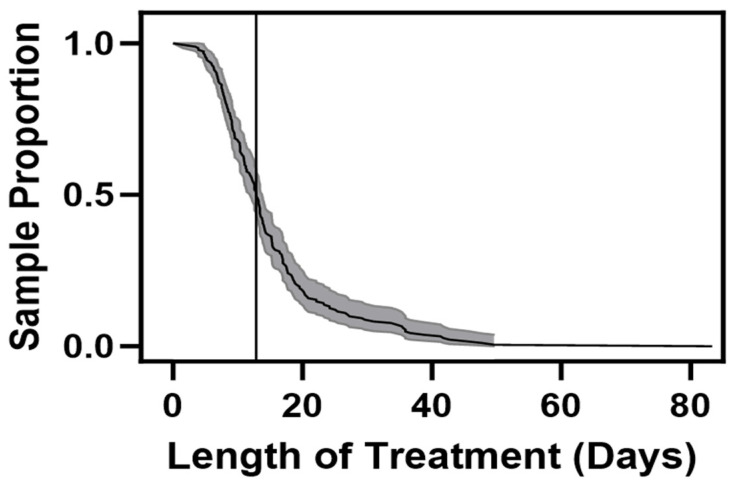
The y-axis represents the proportion of infants plotted against the duration of treatment (x-axis) in days. The vertical line drawn to the left of day 20 indicates the median duration of treatment for all the infants in the study sample.

**Figure 2:**
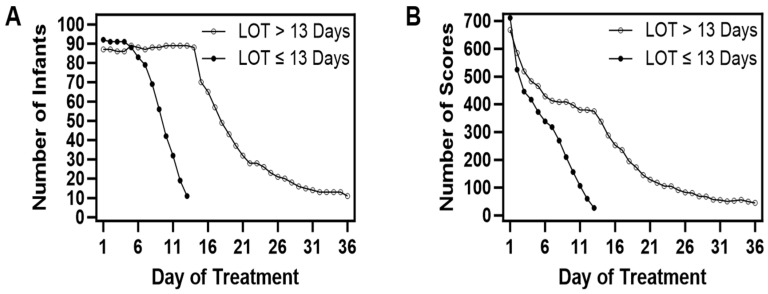
In panel (**A**)**,** the decreasing number of infants in the sample as the LOT increased are shown, with separate plots for the group with a LOT ≤ 13 days or >13 days. Panel (**B**) shows the number of Finnegan Scores of treated infants analyzed per day, with plots separated by LOT ≤ 13 days or >13 days.

We utilized data from a total of 182 unique infants having a median LOT of 12.86 days (Figure 1). Rounding to 13 days as our cutoff for creating treatment groups for comparison, one infant had a LOT between 12.86 and 13 days, and therefore 92 infants were treated for fewer than 13 days, and 90 were treated for more than 13 days. The number of infants and the total number of FS contributed per day are given in Figure 2A,B, respectively.

### 3.2. Mean FS and Prediction of the LOT

**Figure 3:**
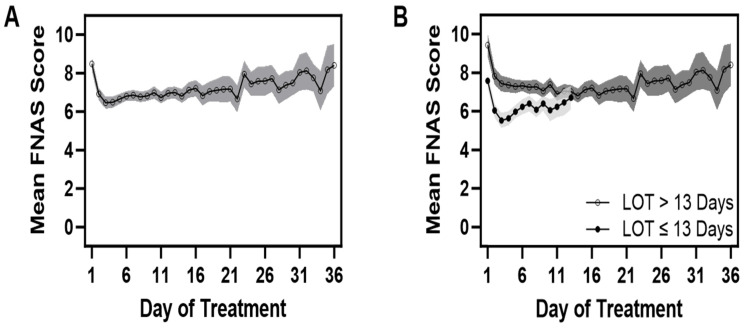
(**A**,**B**) The means (95% CI) of the Finnegan scores (FNAS Score) are plotted over time (duration or day of treatment) for all infants in the sample in panel A, while in panel B, the means (95% CI) of the Finnegan scores are plotted separately for those with a LOT ≤ 13 and a LOT > 13 days.

Overall mean FS decreased from the 1st to the 3rd day of treatment, followed by a slight increase, and stayed relatively constant through the first 13 days regardless of the duration of treatment. Figure 3A provides plots of estimated daily mean FS scores for all infants. The mean scores were lower in the group treated for less than 13 days (Figure 3B), while those requiring longer a duration of treatment beyond 13 days showed sporadic increases in mean scores and tended to be of greater value. However, the decreases in scores within the first 3 days of treatment did not strongly predict the LOT. For any of the mean scores on each of the first three days of treatment, Pearson’s correlations with the natural log of the LOT ranged from 0.41 to 0.43 and were statistically significant (*p* < 0.001). Linear regression results show that, once mean scores on day 1 and day 3 were accounted for, day 2 mean scores were neither statistically (*p* > 0.05) nor clinically (change in R^2^ of ≤0.01) significant. Models incorporating both day 1 and day 3 mean scores as predictors (*p* < 0.05) resulted in R^2^ values ranging from 0.23 to 0.29, *p* < 0.05.

### 3.3. FNAST Item Prevalence and LOT

In general, as mean FS decreased in the first three days of treatment, most notable drops in prevalence in many items were observed. Subsequently, prevalence varied across items; some with sporadic increases over time, while some decreased or had no apparent pattern. Specific clinical signs showed sporadic prevalence among infants with an extended need for treatment.

**Figure 4:**
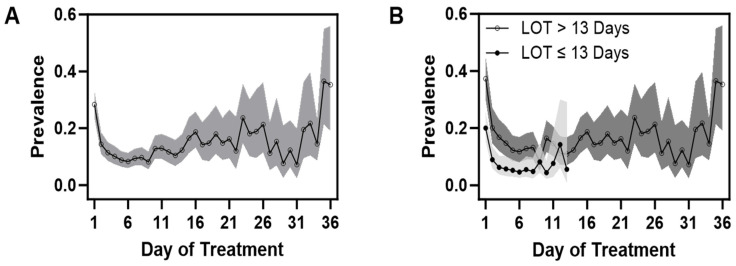
(**A**,**B**) Daily mean prevalence (95% CI) of excessive or continuous high-pitched crying and/or sleep < 1 h in all infants (**A**) and when infants were grouped as to length of treatment (**B**). Note the sporadic increases in prevalence in infants with a LOT > 13 days.

Among CNS signs, the items cry and/or sleep showed dynamic and sporadic changes, as shown in Figure 4A,B. The initial prevalence of continuous high-pitched crying (Supplemental Figure S1A–D) was greater than 25% and dropped to 10% by the third day, with a more marked decrease in those with a short LOT. About 2 weeks after the start of treatment, prevalence increased to about 15%, with slight variation. Sleep duration (Supplemental Figure S2A–F) less than one or two hours after feeding followed a similar pattern as cry and with sporadic increases in prevalence with a longer LOT.

The other CNS signs, i.e., increased tone, hyperactive Moro reflex, and tremors when disturbed, had the greatest prevalence and were estimated initially at 95%, 75%, and 85%, respectively, but were not associated with the LOT. (Supplemental Figure S3A–F). Undisturbed tremors, myoclonic jerks, and generalized convulsions had a very low prevalence or were extremely rare.

**Figure 5:**
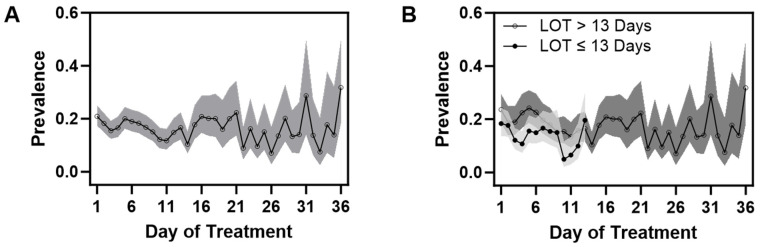
(**A**,**B**) Daily prevalence (95% CI) of loose or watery stools and/or regurgitation or projectile vomiting in all infants over the course of treatment (**A**) and in infants separated by shorter versus longer length of treatment group (**B**).

As to GI signs, the initial prevalence of regurgitation or projectile vomiting of around 10% and loose or watery stools of around 15% demonstrated small decreases initially (Figure 5A,B and Supplemental Figure S4A–D). However, after 2 weeks, those still requiring treatment showed sporadic increases, having prevalences that were close if not higher than the initial prevalence. Excessive sucking and poor feeding (Supplemental Figure S5A–D) followed similar patterns in trajectories; these were more prevalent initially (20%), but subsequent peak increases were at values lower than at the initiation of treatment in those with a longer LOT.

Among metabolic, vasomotor, and respiratory (MVR) signs, initial prevalence varied from around 3% to 35%—lowest with sweating and highest with mottling. These two signs showed either slight or marked but steady increase over the course of treatment, with sporadic increases after two weeks (Supplemental Figure S6A–D). The prevalence of tachypnea and fever (Supplemental Figures S6E,F and S7A,B) decreased slightly at the initial days of treatment, followed by an increase in the first week, and plateaued thereafter; sporadic increases were also evident later through treatment duration (Supplemental Figures S6D and S7B).

## 4. Discussion

Studies have associated certain items in the FNAST with the need to start treatment [14,22]. Through the use of the FNAST in monitoring infants with NAS, we were able to extend our findings to the characterization of each of the many withdrawal signs as they relate to the LOT. We noted the heterogeneity in the prevalence trajectories of these signs, which became prominent with the longer duration of treatment. The mean FS decreased initially from day one to day three day of treatment, then leveled out to a mean FS score of around seven at least through the first two weeks of treatment. In infants needing prolonged treatment, this initial overall improvement was followed by sporadic changes in the presentation of CNS (sleep and cry) and GI (regurgitation/vomiting and loose stools) signs. Thus, the improvement in withdrawal signs noted within a few days of pharmacological treatment initiation was a weak predictor of the LOT.

The changes in mean FS during the first few days into treatment were consistent with the decreased prevalence of most of the withdrawal signs, except for the MVR disturbances (fever, mottling, sweating, nasal stuffiness, sneezing, and increased respiratory rate), which were more common than other signs but were likely to increase over the course of treatment with less variability in prevalence. Although the prevalence of each MVR sign increased, the contribution of each of these less severe signs to the FS, if present, was small (one point). On the other hand, signs such as prolonged crying and decreased sleep duration contributed two to three points to the FS. Therefore, changes in treatment dosing in the management of NAS based solely on total FNAST scores as done for treatment initiation would discount the impact of the severity and sporadic resurgence of certain signs. It is vital that the treatment monitoring of NAS not be based solely on the total FNAST scores.

Considering the interest in using an alternative tool rather than the FNAST, it would make sense that such a tool would include the items that notably change in prevalence over time. Skin conductance [19], infant pupillary diameter [20], and acoustic characteristics of the infant cry [21], while potentially useful in identifying exposure to substances or need for pharmacological management, do not monitor for the pertinent manifestations showing resurgence during prolonged treatment. It appears that the shortened FNAST tools of Gomez et al. and Chervoneva et al. provide an attractive option in the clinical setting, not only in determining a need to initiate treatment but also for monitoring NAS manifestations during treatment [13,15]. Alternatively, there has been an increased interest in using ESC in infants with NAS [34,35,36,37]. Unlike the FNAST-based shortened tools, ESC does not address the manifestations we found pertinent over time while monitoring therapy; thus, its use may fail to identify items of clinical importance. Furthermore, while this approach has gained popularity, long-term outcome studies are still needed, and the tool may not be applicable for the clinical monitoring of all populations [37,38].

We can only speculate on the possible underlying basis of our findings. Morphine is commonly used as the drug of choice to treat NAS [23], with clonidine or phenobarbital as an adjunct medication if morphine alone cannot control withdrawal signs [39,40]. Morphine was the primary treatment in our NAS patients. When considering the infants who required medications to treat withdrawal signs for greater than the median 13 days in our study, there is a possibility that the resurgence of symptoms may have been related to opioid tolerance, and improvement in manifestations would be thereby noted when increasing the morphine dose [41]. Alternatively, rather than withdrawal itself, an opioid effect such as in hyperalgesia, a decrease in dose may be an option [41,42,43]. Other than tolerance or withdrawal, differences in drug clearance due to organ maturation may contribute to these differences in the LOT [44,45]. Furthermore, pharmacogenetic variability in genes mediating drug transport, metabolism, and response could be involved [46,47,48,49,50]. Opioids affect multiple organ systems, and, for example, a high number of µ-receptors are expressed on neurons of the enteric nervous and GI systems [51]. It is possible that what we have observed could be related to the opioid effect on the histamine system and the microbiome [52]. We speculate that the regurgitation or vomiting may be related to opioid-induced vomiting with suggested mechanisms of histamine release, activation of the mu-receptors in the chemoreceptor trigger zone, and delayed gastric emptying [52,53], suggesting that patients may benefit from Histamine-1 and Histamine-2 receptor blockers. Interestingly, Maguire et al. suggested that opioid exposure during gestation and the development of NAS at birth may lead to a dysbiotic gut [54]. This concept was also raised by Sealschott and colleagues [55]. Consistent with crying as a manifestation in NAS, prolonged crying is also noted in colicky babies with a dysbiotic microbiome [56]. Thus, it is possible that the opioid effects on the gut–brain axis could explain some of our findings [54,56,57]; these speculations warrant further investigation.

As for clinical implications, our findings support the importance of optimizing the treatment management of NAS in the neonatal period. Attributing the signs during prolonged treatment strictly to substance withdrawal may overlook the possible biological factors that may need to be addressed by interventions other than dose titration. Maternal emotional or psychological situations while caring for the infant during in-hospital treatment could affect the infant’s neurobehavioral state. The CNS signs of prolonged crying and sleep disturbances may render caring for an infant with NAS stressful, which may affect caretaker–child interaction and bonding. GI signs, if severe, may lead to sub-optimal growth and failure to thrive.

Parents or caretakers need education regarding the possibility of resurgence or persistence of manifestations post-discharge especially after a short hospital stay, a common primary outcome for many NAS quality care initiatives. Unfortunately, the length of stay or LOT may not be an ideal outcome measure. Multiple issues have been raised in NAS beyond discharge even as early as the first year of life, such as the risk of sudden unexpected deaths, maltreatment, frequent hospitalization, and emergency room visits [58]. The encounter between the clinician and child in the newborn period provides early opportunities not only to improve the management of NAS but also to promote the plan of safe care, enhance post-discharge outcomes, and minimize and/or prevent morbidities from a multifactorial interplay of risk factors [5,58].

Our study had limitations. First, the data were collected retrospectively at a single center. Second, there was increased variability in the prevalence of signs over time that might be explained by attrition after about half of the infants had completed treatment. We were not able to analyze the effects of the maternal use of other drugs or types and quantities of opioids, as the information was not included in the deidentified dataset used in this study. Lastly, the LOT may have been affected by maternal lifetime experiences, access to treatment [59,60,61], and other biological and genetic factors [62,63,64,65,66,67] that were not addressed in this study.

## 5. Conclusions

From our retrospective study, the finding of the sporadic recurrence of signs over time is new and could pave the way for future research on the effects of prenatal opioid exposure that may also be heightened by postnatal treatment exposure. Furthermore, it is important to fully understand why and how infants with NAS differ in response to pharmacologic treatment. More attention needs to be directed to individual withdrawal signs, any relationship between signs, the variable trajectory from one sign to the other, and the careful consideration for possible opioid effects when observing a resurgence of these signs in the course of treatment. The use of a comprehensive tool such as the FNAST, beyond the calculation of total scores, offers the advantage of a careful and thorough assessment of infants with NAS, before, during, and at the completion of treatment. Until we have a better understanding of why and when the resurgence of certain manifestations occurs during treatment, it may not be time to abandon the FNAST. Lastly, our findings do call for future studies to explore prenatal and postnatal opioid exposure effects on the gut–brain axis and microbiomes. Research on the underlying mechanisms that may explain CNS and GI manifestations in NAS has the potential to discover treatment that will prevent or mitigate the manifestations related to opioid dysbiosis.

## Data Availability

The data presented in this study are available on request from the corresponding author. The data are not publicly available due to privacy concerns.

## Supplementary Materials

The following supporting information can be downloaded at: https://www.mdpi.com/article/10.3390/children11020203/s1. Figure S1: Trajectory of high-pitched crying and continuous crying for all infants and by treatment groups; Figure S2: Trajectory of sleep < 1 h, <2 h, and <3 h overall and by treatment groups; Figure S3: Prevalence of tremors when disturbed, tremors when undisturbed, and increased tone in all infants and by treatment groups; Figure S4: Prevalence of regurgitation/vomiting and loose/watery stools in all infants and by treatment groups; Figure S5: Prevalence of excessive suck and poor feeding for all infants and by treatment groups; Figure S6: Prevalence of sweating, mottling, and increased respiration for all infants and by treatment groups; Figure S7: Prevalence of fever in all infants and by treatment groups.
