# Supplementary material for: Neonatal Abstinence Signs during Treatment: Trajectory, Resurgence and Heterogeneity
Source: Children (Basel). 2024 Feb 5;11(2):203. doi: 10.3390/children11020203 (PMC10887053; doi:10.3390/children11020203)
Supplement: Supplementary file 1 [file children-11-00203-s001.zip › children-2845364-supplementary.pdf]

Figure S1A-D: Trajectory of high-pitched crying and continuous crying for all infants and by treatment groups.

Figure S2A-F: Trajectory of sleep < 1 hour, < 2 hours, and <3 hours overall and by treatment groups.

Figure S3A-F: Prevalence of tremors when disturbed, tremors when undisturbed, and increased tone in all infants and by treatment groups.

Figure S4A-D: Prevalence of regurgitation/vomiting and loose/watery stools in all infants and by treatment groups.

Figure S5A-D: Prevalence of excessive suck and poor feeding for all infants and by treatment groups.

Figure S6A-F: Prevalence of sweating, mottling, and increased respiration for all infants and by treatment groups.

Figure S7A-B: Prevalence of fever in all infants and by treatment groups.

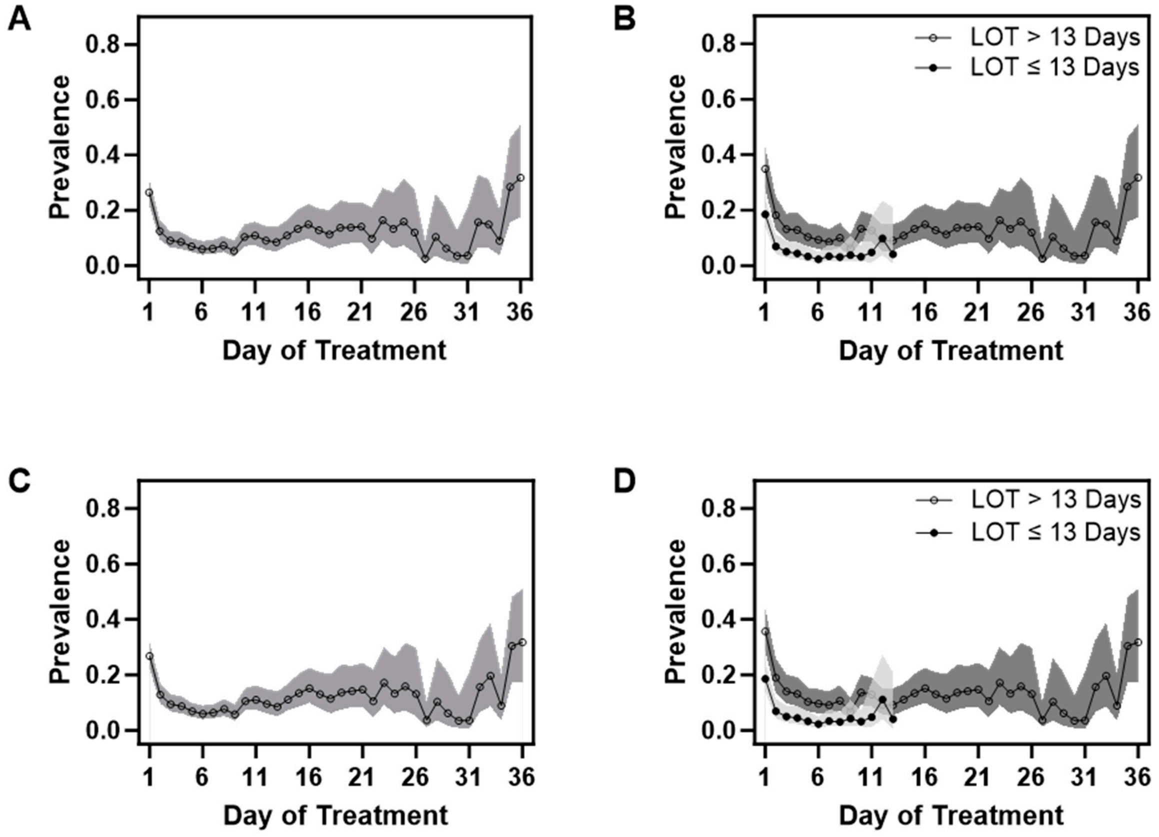

Figure S1A-D. Figure S1 (Panel A) shows the daily mean prevalence (95% CI) of high-pitched crying in all infants throughout the duration of the treatment. Panel B shows the daily mean prevalence (95% CI) in infants by LOT ≤13 days and >13 days. Note that, after 2 weeks of treatment, prevalence increased with sporadic peaks in those with LOT >13 days. Panel C shows the prevalence (95% CI) of excessive continuous crying in all infants, while Panel D shows the prevalence separating the plots for those with a LOT ≤13 days and those treated >13 days.

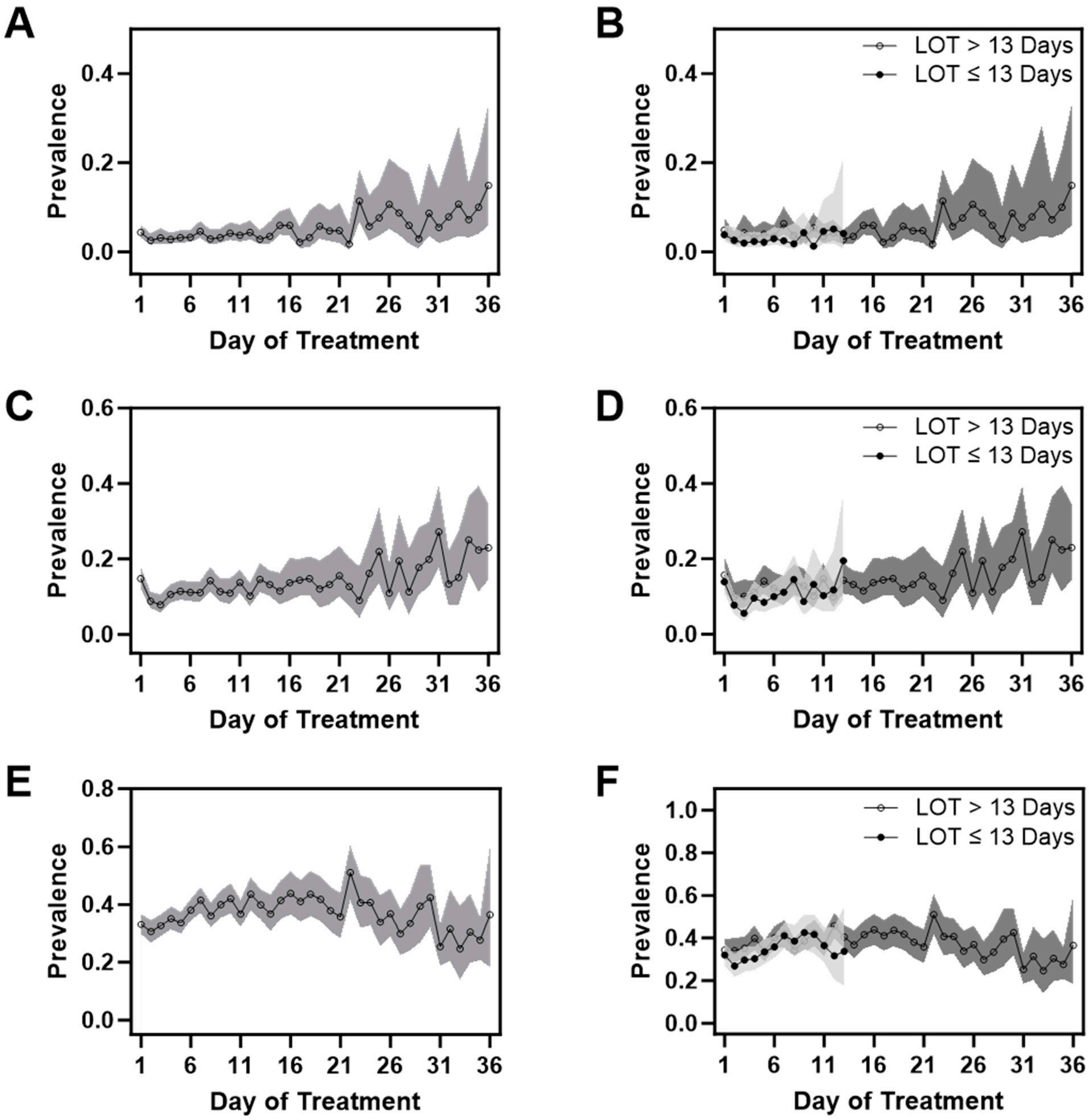

Figure S2A-F. Daily prevalence of sleep <1 hour (Panel A), <2 hours (Panel C), and <3 hours (Panel E) for all infants in this study. Panels B, D, and F show the prevalence on a given day for sleep <1 hour, <2 hours, and <3 hours, respectively, with separate plots for those with a LOT ≤13 days and those treated for >13 days.

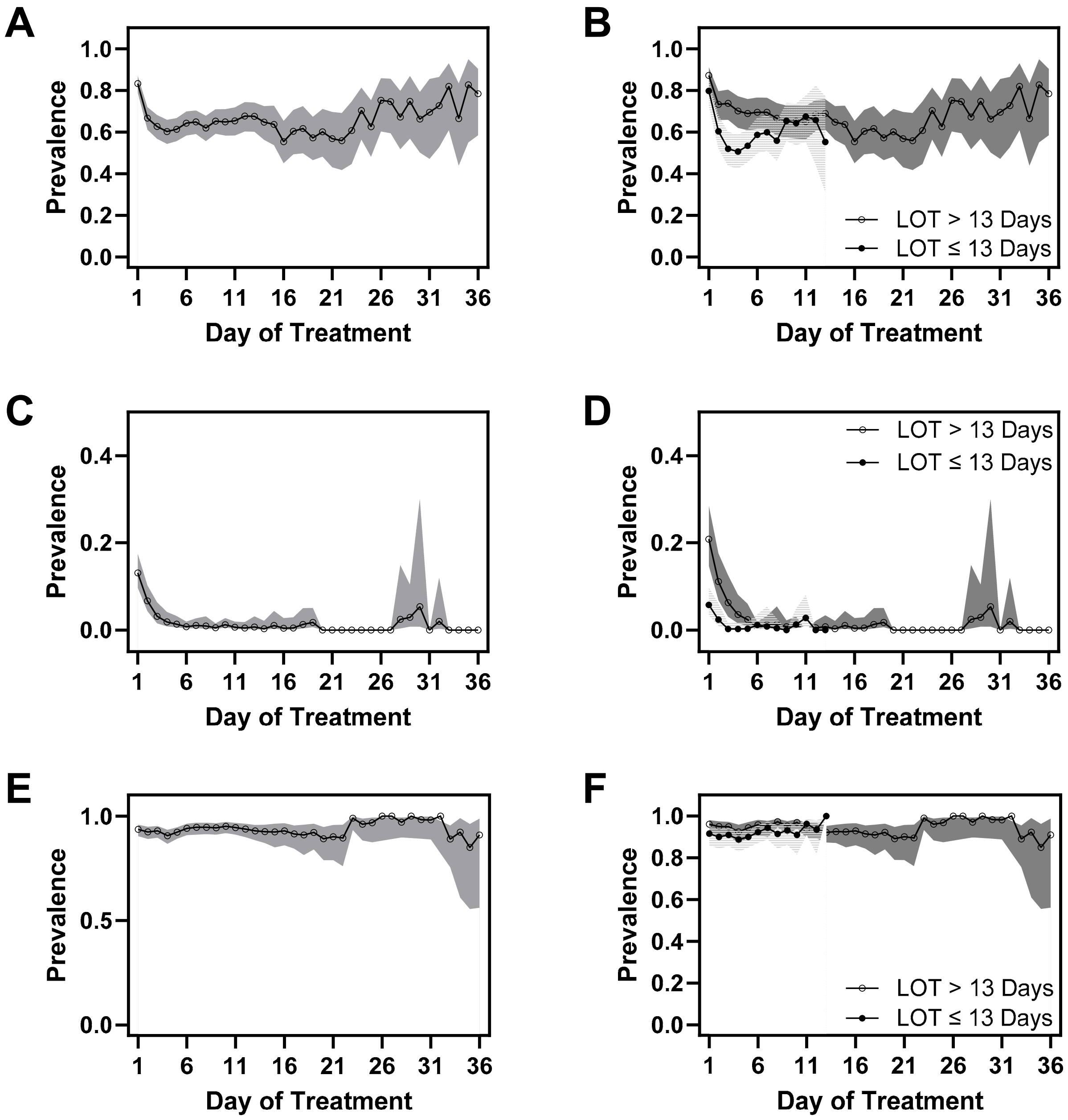

Figure S3A-F. Panel A illustrates the prevalence of tremors when disturbed for all treated infants while Panel B shows the separate plots for those treated for 13 days or less and those with LOT >13 days. Panel C shows the daily prevalence of undisturbed tremors for the whole sample, while Panel D illustrates the daily prevalence with the short LOT compared with the longer LOT (>13 days). Note that Panel E shows the very high prevalence of increased tone or hypertonia, and in Panel F, very little difference is noted when the duration of treatment is ≤13 days or >13 days.

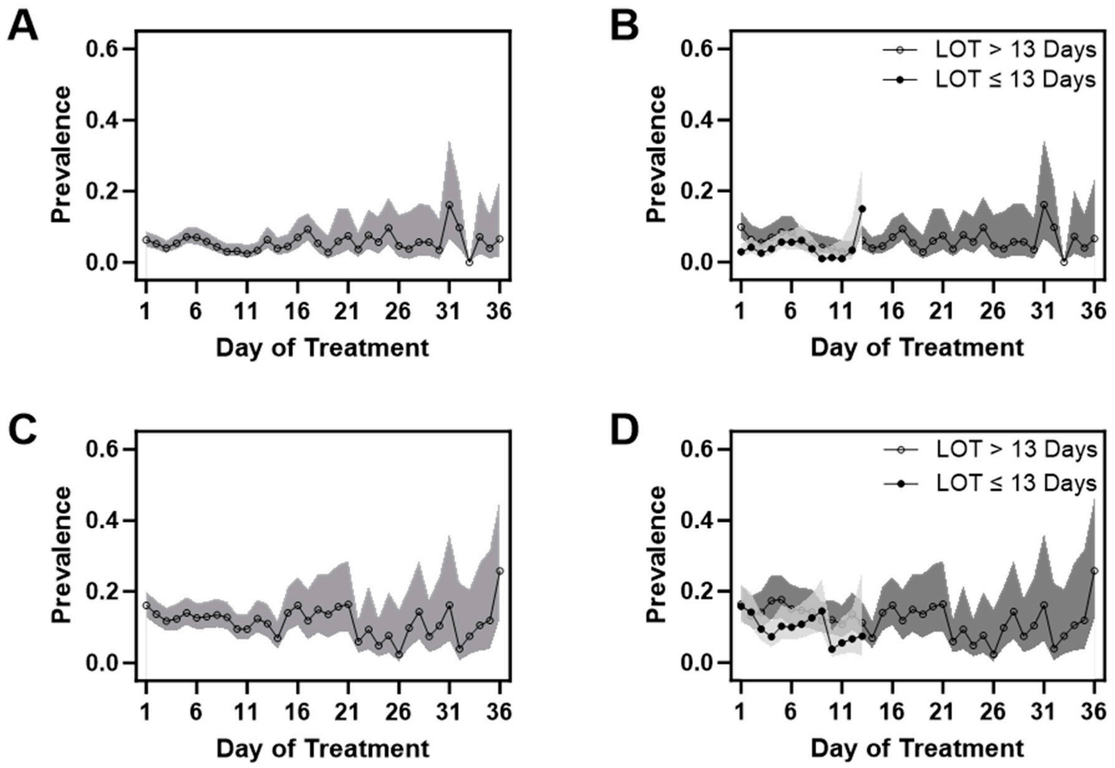

Figure S4A-D. Panel A illustrates the prevalence of regurgitation or vomiting noted in all treated infants. Intermittent peaks of prevalence occurred after 2 weeks of treatment, mostly noted in infants with a prolonged LOT. In Panel B, the prevalence plots are separated between the treated groups. Prevalence decreased during the first 2 weeks of treatment, but the group treated for >13 days had a resurgence of these signs, with prevalence as high or even higher than the initial prevalence noted at the start of treatment. Panels C and D, respectively, illustrate the daily prevalence during treatment for all infants and by the LOT for the manifestations of loose to watery stools. Sporadic increases in prevalence occurred after 2 weeks and, at times, with peaks higher than prevalence during the first few days of treatment.

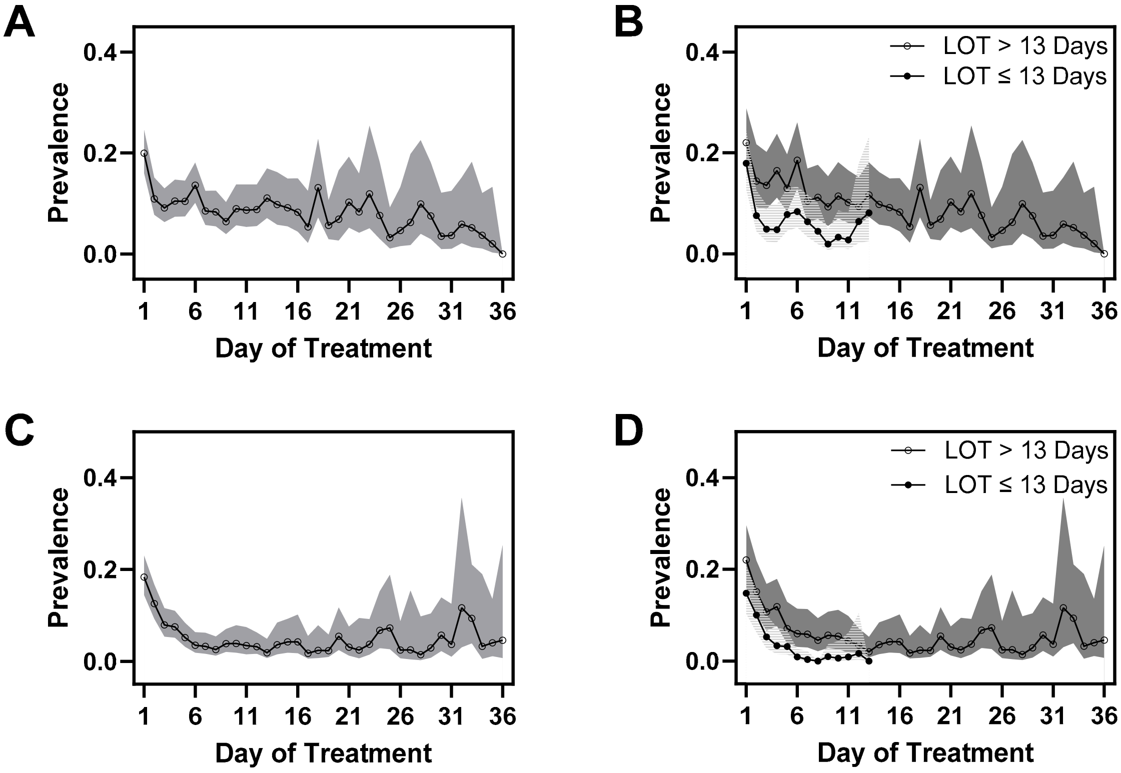

Figure S5A-D. Panel A shows the prevalence of excessive suck for all infants on a given day of treatment. In Panel B, the group treated for ≤13 days showed stable improvement over the course of treatment while those treated for a longer duration showed a decreasing trend in daily mean prevalence with intermittent peaks, trending lower, however, than the initial prevalence in most infants. Panel C shows the decreasing trend in the prevalence of poor feeding for the whole sample. In Panel D, those treated for longer than 13 days show sporadic increases in prevalence with peaks appearing to increase after the 3rd week of treatment.

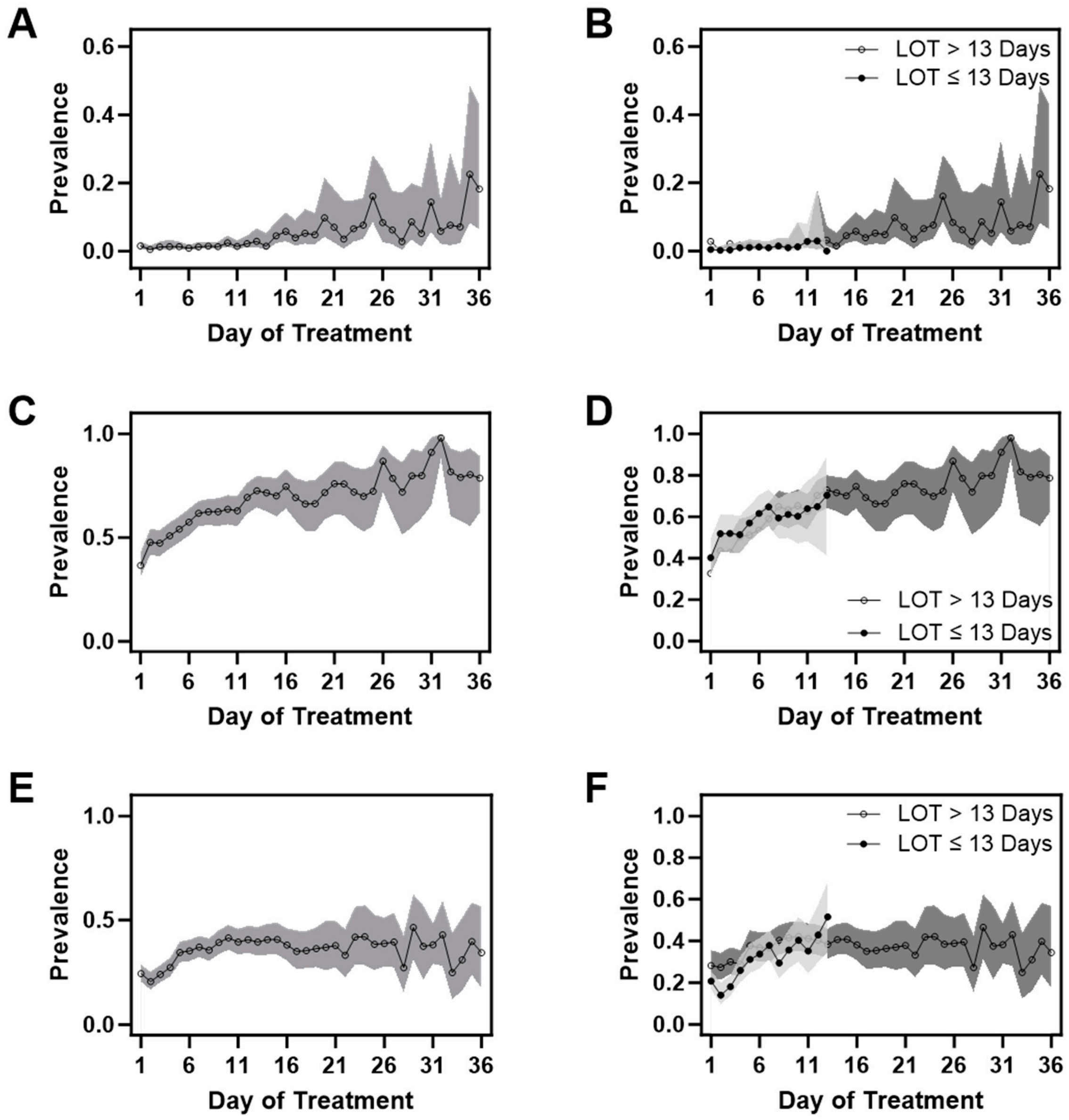

Figure S6A-F. The figure shows the prevalence for all cases of sweating (Panel A), mottling (Panel C), and increase in respiratory rate (Panel E). These signs increased with each day of treatment; however, further increase after two weeks was noted only with sweating and mottling, with intermittent peaks in those with a LOT >13 days (Panels B and D). The increased respiratory rate sign demonstrated increasing prevalence during the first week (Panel E) and appeared to plateau thereafter, with intermittent peaks after 3 weeks of treatment (Panel F) in those treated for >13 days.

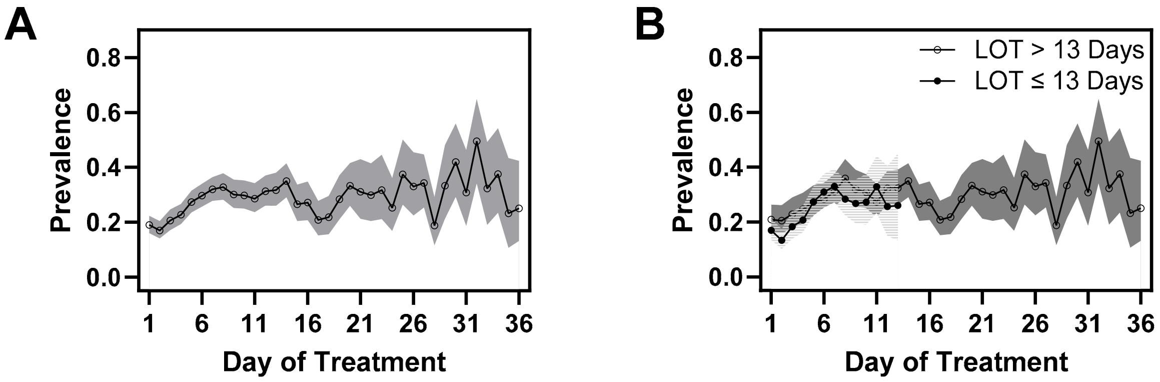

Figure S7A-B. Panel A shows that the prevalence of fever increased and reached almost double the pre-treatment value after 1 week of treatment in all infants, followed by intermittent increases in prevalence that became noticeable by the 3rd week of treatment in those with a LOT >13 days. (Panel B).
